# siRNA-Inhibition of TIGAR Hypersensitizes Human Papillomavirus-Transformed Cells to Apoptosis Induced by Chemotherapy Drugs that Cause Oxidative Stress

**Published:** 2021-05-31

**Authors:** Lacin Yapindi, Brenda Y. Hernandez, Robert Harrod

**Affiliations:** 1 Laboratory of Molecular Virology, Department of Biological Sciences, The Dedman College Center for Drug Discovery, Design and Delivery, Southern Methodist University, Dallas, Texas, 75275-0376, United States; 2 University of Hawaii Cancer Center, Honolulu, Hawaii, 96813, United States

**Keywords:** Oxidative stress, Cervical cancer, Chemotherapy, DNA-damage, Apoptosis

## Abstract

The high-risk subtype Human Papillomaviruses (hrHPVs), including HPV16, HPV18, HPV31, HPV33, and HPV45, infect and oncogenically transform epithelial cells and cause squamous cell carcinomas and adenocarcinomas associated with the development of cervical cancer and subsets of vulvar, vaginal, penile, and anogenital cancers, as well as head-and-neck oropharyngeal carcinomas which often have poor clinical prognoses. Many cancers have been shown to contain elevated levels of the TP53-Induced Glycolysis and Apoptosis Regulator (TIGAR)-a glycolytic enzyme and antioxidant effector which frequently correlates with an aggressive tumor phenotype and serves as a determinant of therapy-resistance. We therefore tested whether siRNA-inhibition of TIGAR protein expression could sensitize HPV18-transformed HeLa cells to genotoxic chemotherapy agents (i.e., cisplatin, etoposide, doxorubicin, and 4-hydroxycyclophosphamide) that induce oxidative stress and DNA-damage. Here we demonstrate that the siRNA-knockdown of TIGAR hypersensitized HeLa cells to low, otherwise sub-inhibitory concentrations of these drugs and markedly induced cellular apoptosis, as compared to a scrambled RNA (scrRNA) oligonucleotide negative control or a non-transformed immortalized human fibroblast cell-line, HFL1. Importantly, these findings suggest that therapeutically inhibiting TIGAR could hypersensitize hrHPV+ cervical tumor cells to low-dosage concentrations of chemotherapy drugs that induce oxidative DNA-damage, which could potentially lead to more favorable clinical outcomes by reducing the adverse side-effects of these anticancer medications and making them more tolerable for patients. Our studies have further shown that siRNA-inhibition of TIGAR sensitizes HPV18+ HeLa cells to apoptosis induced by 4-hydroxycyclophosphamide-a DNA-alkylating agent these cells were reported to have resistance to, alluding to another possible benefit of targeting TIGAR in combinatorial treatment strategies against virus-induced cancers.

## INTRODUCTION

It is estimated that approximately 15%-20% of human cancers are caused by oncogenic viruses, such as the human T-cell leukemia virus type-1 (HTLV-1), Epstein-Barr virus (EBV), hepatitis B and C viruses (HBV/HCV), Kaposi’s sarcoma-associated herpesvirus/human herpesvirus-8 (KSHV/HHV-8), and high-risk subtype Human Papillomaviruses (hrHPVs), although the molecular mechanisms by which these oncoviruses deregulate host cellular growth and proliferative pathways remain to be fully elucidated [1–13]. The α- type hrHPVs infect epithelial cells and are etiologically linked to sexually-transmitted genital warts and cervical dysplasia and the development of cervical, vulvar, vaginal, penile, anogenital and head-and-neck oropharyngeal malignancies [10–17]. Although at least two multivalent preventative vaccines (i.e., Gardasil and Cervarix) have been developed against the major high-risk subtype HPVs, there continues to be high incidences of cervical and anogenital cancers and oropharyngeal carcinomas; and these vaccines are not indicated for the treatment of pre-existing hrHPV-infections or to prevent disease progression related to hrHPV-associated cancers [18]. In the present study, we have tested whether inhibition of the Tp53-Induced Glycolysis and Apoptosis Regulator (TIGAR) using a targeted small-interfering RNA (siRNA-tigar) could sensitize the HPV18-transformed HeLa cervical adenocarcinoma cell-line to genotoxic chemotherapy agents that induce oxidative stress and DNA-damage in efforts to determine whether TIGAR-mediated antioxidant signaling promotes therapy-resistance in hrHPV-associated cancers [19–24].

The Human Papillomaviruses are members of the *Papovavirus* family (which also includes the structurally-related polyomaviruses) and are small, non-enveloped viruses with icosahedral symmetry and circular double-stranded DNA genomes of approximately 8 kilobases in size [25–28]. There are estimated to be around 200 different low-risk subtype HPVs (e.g., HPV1, HPV2, and HPV4) which cause benign hyperproliferative epithelial lesions, including skin warts or plantar warts [26,29]. By contrast, HPV16, HPV18, HPV31, and HPV45 are the most common high-risk subtypes associated with the development of epithelial malignancies, including cervical carcinomas and head-and-neck cancers; and it has been estimated that 90% of cervical cancers are positive for HPV [10]. The viral oncoproteins E6 and E7 are primarily responsible for the cell-immortalizing and transforming activity of the hrHPVs. The E7 oncoprotein promotes S-phase entry and cellular proliferation through its interactions with the retinoblastoma (Rb) tumor suppressor and degrades Rb and disrupts the sequestration of nuclear E2F complexes by Rb-E2F [30–32]. However, using a transgenic mutant Rb knock-in mouse model that expresses a mutant form of the Rb protein defective for binding E7, Balsitis et al. [33] demonstrated that the non-Rb targets of E7 likely contribute to viral carcinogenesis leading to the development of cervical cancers. The CR3 region of the E7 oncoprotein interacts with the octamer-binding transcription factor-4 (Oct4) [34] which has been shown to contribute to pluripotency and cellular reprogramming, together with c-Myc, Sox2, and Klf4, and is negatively regulated by Rb [35]. The HPV E6 oncoprotein has been shown to cooperate with the c-Myc oncogene and induces cellular immortalization by activating the promoter of the htert gene [36,37]. Liu et al. have further demonstrated that E6 physically interacts with the hTERT protein [37]. Moreover, both E6/E7 enhanced the expression of Oct4 in HPV+ cervical cancer cells [34]. The E6 oncoprotein also destabilizes and induces the degradation of the p53 tumor suppressor through recruitment of the E3 ubiquitin ligase, E6-associated protein (E6AP), and inhibits p53-dependent apoptosis in HPV-transformed cells [38–40]. Jha et al. have also shown that E6 inhibits the acetylation of p53 on lysine residue K120 by the Tat-interacting protein of 60 kDa (TIP60) and thereby prevents the induction of p53-dependent apoptotic genes [41]. Nevertheless, the p53 protein is detectably expressed in most hrHPV+ cervical carcinoma cell-lines; and several studies have demonstrated the remaining p53 in these cells is transcriptionally active [42–44]. It is intriguing that, although the p53 tumor suppressor protein is destabilized and functionally impaired in its ability to induce apoptosis as a result of the viral E6 oncoprotein, p53 is rarely mutated in hrHPV+ cancers-which suggests p53-regulated target genes may somehow contribute to viral carcinogenesis [45].

The TIGAR protein is a p53-regulated 2,6-bis-fructose-phosphatase that prevents the accumulation of damaging mitochondrial Reactive Oxygen Species (ROS) in response to oncogenic proliferative signaling by increasing the levels of free NADPH and the antioxidant effector, reduced glutathione [19,20]. TIGAR has been shown to target the mitochondrial outer membrane under conditions of hypoxic stress and glucose-deprivation, associated with its interactions with hexokinase-2 (HK2) and requiring the activation of hypoxia-inducible factor-1 alpha (HIF-1α) signaling [46]. We and others have demonstrated that TIGAR is highly expressed in many different human cancers and frequently correlates with an aggressive disease phenotype, therapy-resistance, and poor clinical outcomes [47–61]. The overexpression of TIGAR was also shown to serve as a prognostic indicator of advanced tumor growth and invasiveness in cervical cancer patients [62].

Here we demonstrate that the TIGAR protein is highly expressed in primary hrHPV+ cervical carcinoma clinical isolates, and that the siRNA-inhibition of TIGAR expression hypersensitizes HPV18-transformed HeLa cervical adenocarcinoma cells to low-dose concentrations of genotoxic chemotherapy drugs that cause oxidative stress and DNA-damage. These findings suggest that targeting the antioxidant effector TIGAR through combinatorial treatment strategies could represent a plausible therapeutic approach against oncogenic viruses and viral-induced malignancies.

## MATERIALS AND METHODS

### Cell-lines, culture methods, and siRNA transfections

The HPV18-transformed HeLa cervical adenocarcinoma cell-line (American Type Culture Collection (ATCC) No. CCL-2) was grown in 75 cm^2^ plastic poly-lysine-coated tissue culture flasks (Greiner Bio-One) in Dulbecco’s Modified Eagle’s Medium (DMEM; ATCC No.30-2002), supplemented with 10% heat-inactivated fetal bovine serum (FBS; Biowest), 100 U/ml penicillin, 100 μg/ml streptomycin-sulfate, and 20 μg/ml gentamycin-sulfate (Life Technologies), at 37°C under 5% CO_2_, in a humidified incubator. The immortalized human fibroblast HFL1 cell-line (ATCC No. CCL-153) was cultured in F-12K Medium (ATCC No.30-2004), supplemented with 10% heat-inactivated FBS, 100 U/ml penicillin, 100 μg/ml streptomycin-sulfate, and 20 μg/ml gentamycin-sulfate, and incubated at 37°C under 5% CO_2_.

For the transfection experiments with small-interfering RNAs (siRNAs), the HPV18-transformed HeLa cells and HFL1 fibroblasts were washed 2x with serum-free medium and trypsinized using a 0.25% Trypsin-EDTA solution (ATCC No. 30-2101) and pelleted by centrifugation in 15 ml polypropylene conical tubes (Corning) for 7 min at 260 x g and 4°C in an Eppendorf model 5810R refrigerated tabletop centrifuge with a swinging bucket rotor. The cells were then resuspended in complete medium, supplemented with serum and antibiotics, and 1.5 × 10^5^ cells were plated and grown on poly-L-lysine-treated 18 mm^2^ glass coverslips (Fisher Scientific) in 35 mm^2^ tissue-culture dishes (Greiner Bio-One) and transfected with 25 ng of a 2’-O,4’aminoethylene bridged nucleic acid (BNA) modified as si RNA-TIGAR oligonucleotide (5’-AUAUACCAGCAGCUGCUGC-3’) with phosphorothioate linkages at the 5’ and 3’ ends, or a 2’O-methyl-uridine-modified scrambled RNA (scrRNA) oligonucleotide (5’UUACCGAGACCGUACGUAU-3’) with terminal 5’ and 3’ phosphorothioate linkages as a negative control (Biosynthesis). All transfections were carried out using HiPerFect transfection reagent (Qiagen) as recommended per the manufacturer’s protocol.

### SDS-PAGE and immunoblot analysis of TIGAR protein expression in drug-treated HPV18+ HeLa cells

To determine whether the chemotherapy agents, cisplatin, etoposide, doxorubicin, or 4-hydroxycyclophosphamide, might affect the expression of the TIGAR protein, 2 × 10^5^ HPV18-transformed HeLa cells were plated in each well and grown on 6-well tissue-culture plates (Greiner Bio-One) in DMEM, supplemented with 10% heat-inactivated FBS, 100 U/ml penicillin, and 100 μg/ml streptomycin-sulfate, in a humidified incubator at 37°C under 10% CO_2_. The media was later removed, and the cells were washed 2x with serum-free DMEM, and complete medium (without antibiotics) was added that contained either cisplatin (200 μM), etoposide (200 μM), doxorubicin (1 μM), 4-hydroxycyclophosphamide (200 μM), or 1% Dimethyl Sulfoxide (DMSO; Sigma-Aldrich) as a negative control. The chemotherapy compounds were all purchased from Sigma-Aldrich and diluted and prepared as stock solutions in 100% DMSO at an initial concentration of 100 μM. The complete media containing the chemotherapy drugs were sterilized by vacuum filtration using a disposable 0.2 μm unit (Corning) before adding them to the cells. The treated cultures were incubated at 37°C under 10% CO_2_ for another 24 hrs. The cells were subsequently harvested by scraping, pelleted by centrifugation in 15 ml polypropylene conical tubes (Corning) for 7 min at 260 x g at 4°C, and resuspended in 80 μl of 1x Lysis Buffer (Promega). Cell lysis was performed by repeatedly freeze-thawing the samples on dry-ice and then passaging them through a 27-gauge syringe needle. The samples were clarified by centrifugation for 2 min at 5000x g at 4°C, mixed with 2x Laemmli Samples Buffer that contained 2-mercaptoethanol (Biorad Laboratories), and then heat-denatured at 95°C for 5 min. The proteins were resolved by Sodium Dodecyl Sulfate-Polyacrylamide Gel Electrophoresis (SDS-PAGE) using a Tris-glycine 12.5% polyacrylamide running gel and a 4% stacking layer. The samples were eventually transferred to a Protran BA83 0.2 μm nitrocellulose membrane (Whatman) using a model TE 77 PWR semi-dry blotting unit (Amersham Biosciences). The transferred proteins were visualized by staining the membranes with a Ponceau S dye solution (0.1% w/v Ponceau S and 5% glacial acetic acid in distilled deionized water). The membranes were rinsed with distilled deionized water and then incubated in Blocking buffer (3% w/v bovine serum albumin and 0.5% Tween-20 in PBS, pH 7.4) for 1 h on a shaker with gentle agitation. The Blocking buffer was later removed and immunoblotting was performed by incubating the membranes for 2 h in the primary antibodies (i.e., rabbit polyclonal Anti-TIGAR, clone ab2, antibody; Sigma-Aldrich, and goat polyclonal Anti-Actin, clone I-19, antibody; Santa Cruz Biotechnology) which were diluted 1:1000 in Blotto buffer (50 mM Tris-Cl, pH 8.0, 2 mM CaCl_2_, 80 mM NaCl, 0.2% v/v IGEPAL-CA630, 0.02% sodium azide, and 5% w/v nonfat dry milk), and the samples were washed 2x with Blotto buffer for 10 min with gentle shaking, and subsequently incubated in the secondary antibodies (i.e., Horseradish Peroxidase (HRP)-conjugated goat Anti-Rabbit IgG; HRP-conjugated donkey Anti-Goat IgG; Santa Cruz Biotechnology), diluted 1:1000 in Blotto buffer for 1.5 h with agitation. The membranes were then washed 2x with Blotto buffer and once with TMN buffer (100 mM NaCl, 5 mM MgCl_2_, and 100 mM Tris-Cl, pH 9.5) for 10 min each, and developed by chemiluminescence detection using Pierce ECL Western Blot Reagent (Thermo Scientific) and a Chemi Doc Touch imaging system (Biorad Laboratories). The relative levels of TIGAR protein expression were quantified by densitometry analysis and normalized to the amount of Actin protein in each sample.

### Measuring cellular apoptosis

To determine if the siRNA-knockdown of TIGAR expression could sensitize HPV18-tranformed HeLa cells to cytotoxicity induced by chemotherapy agents that cause oxidative DNA-damage, the siRNA-TIGAR (or scrRNA control) transfected cultures were treated with various concentrations of either cisplatin, etoposide, doxorubicin, or 4-hydroxycyclophosphamide for 24 h at 37°C under 5% CO_2_ in a humidified incubator. The samples were then stained using a microscopy-based apoptosis detection kit (BD-Pharmingen) with Annexin V conjugated to Fluorescein Isothiocyanate (Annexin V-FITC) and the nuclear stains: Propidium Iodide (PI) or 4’,6-diamidino-2-phenylindole (DAPI; Sigma-Aldrich), and the relative percentages of apoptotic (i.e., Annexin V-FITC and/or PI-positive) cells per field were quantified by confocal fluorescence-microscopy at 200x magnification by counting triplicate fields. The total numbers of cells were quantified by microscopy using a DIC phase-contrast filter.

### Analysis of TIGAR expression in primary HPV16-infected cervical cancer clinical samples

To assess the expression of the TIGAR protein within the E6-positive cells of HPV+ tumor tissues, primary HPV16-infected cervical carcinoma patient isolates were provided by the Pathology Core and Hawaii Tumor Registry of the University of Hawaii Cancer Center as de-identified, Formalin-Fixed Paraffin-Embedded (FFPE) tissue sections under a protocol approved by the SMU Human Subjects-Institutional Review Board and in accordance with the Declaration of Helsinki principles. The samples were deparaffinized by treating them 2x with xylene for 3 min at room temperature, followed by a 1:1 xylene/ethanol solution for 3 min, 2x treatment with 100% ethanol for 3 min, 95% ethanol for 3 min, 70% ethanol for 3 min, 50% ethanol for 3 min, and finally rinsing them 2x with distilled deionized water. The samples were then incubated in Blocking buffer for 1 h at room temperature with gentle agitation. After removal of the buffer, the tissue sections were incubated with primary antibodies (i.e., goat polyclonal Anti-HPV16 E6 clone N-17 and rabbit polyclonal Anti-TIGAR clone M-209, Santa Cruz Biotechnology), diluted 1:500 in Blotto buffer, for 2 h with gentle agitation. The samples were then washed 2x with Blotto buffer for 10 min and immunostained with appropriate fluorescent secondary antibodies (i.e., Alexa Fluor 488-conjugated AffiniPure donkey anti-goat IgG (H+L), and Rhodamine Red-X-conjugated AffiniPure donkey anti-rabbit IgG (H+L), Jackson ImmunoResearch Laboratories), diluted 1:500 in Blotto buffer, for 1.5 h at room temperature with gentle agitation. The samples were co-stained with a 5 mg/ml solution of DAPI (Sigma-Aldrich) to visualize all cells within the tumor microenvironment and surrounding stroma. Finally, the samples were washed 2x with Blotto buffer and once with PBS, pH 7.4, for 10 min and fluorescence-microscopy mounting medium was added (Kirkegaard and Perry Lab), together with a 22 × 60 mm cover glass. The samples were subsequently analyzed by immunofluorescence-confocal microscopy which was performed on a Zeiss LSM800 instrument using a Plan-Apochromat 20x/0.8 objective lens with 16 averages per image. The relative expression of the TIGAR and HPV16 E6 proteins was quantified and graphically represented using the 2.5 D tool in the Carl Zeiss Microscopy Zen OS software.

### Microscopy

The expression of the TIGAR protein and E6 viral oncoprotein within the HPV16-infected cervical cancer clinical samples by immunofluorescence staining, as well as the detection of cellular apoptosis using Annexin V-FITC/PI (or DAPI), were visualized confocal fluorescence-microscopy on a Zeiss LSM800 instrument using a Plan-Apochromat 20x/0.8 objective lens and ZEN OS software (Carl Zeiss Microscopy). The relative fluorescence-intensities of the Anti-TIGAR-specific (Rhodamine Red-X-positive) and Anti-HPV16 E6-specific (Alexa Fluor 488-positive) signals were graphically quantified using the Zen 2.5D analysis tool (Carl Zeiss Microscopy).

### Data analysis

The statistical significance of experimental data sets was determined using unpaired two-tailed Student’s t-tests (alpha=0.05) and calculated P-values using the Shapiro-Wilk normality test and Graphpad Prism 8.0 software. P-values less than 0.05 were considered to be statistically significant. The error bars represent the SEM from three independent experiments.

## RESULTS

### The TIGAR protein is highly expressed in primary HPV16+ cervical cancer clinical isolates

**Figure 1:**
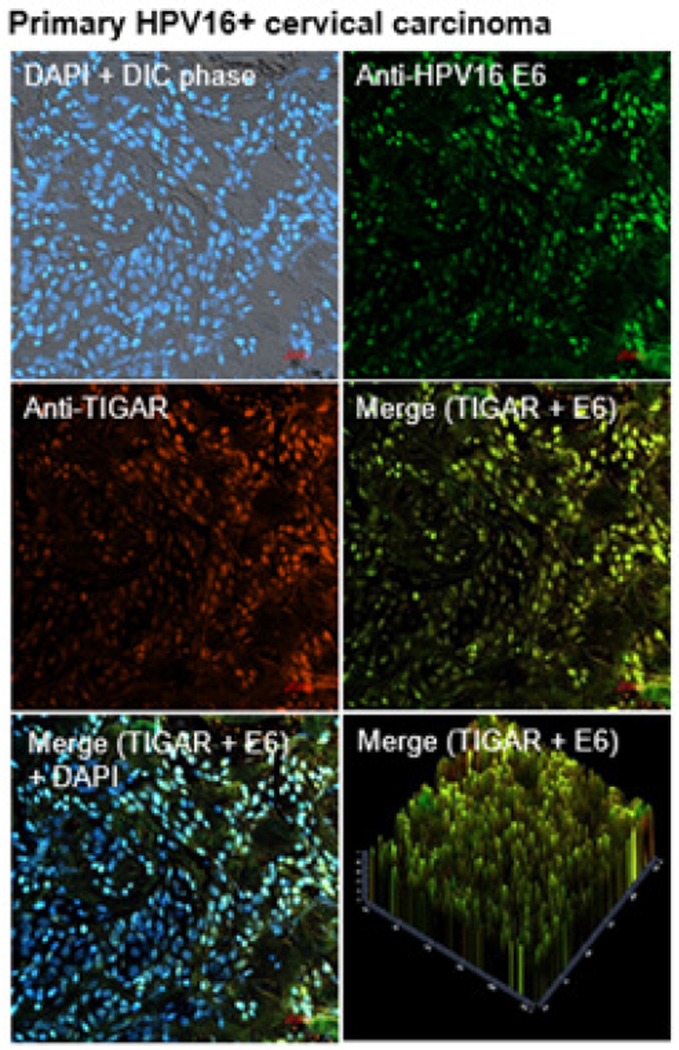
The TIGAR protein is highly expressed in primary HPV16-infected cervical carcinoma clinical isolates. Representative micrographs depict HPV16+ cervical carcinoma tumor sections (FFPE, obtained from the University of Hawaii Cancer Center) that were deparaffinized and immunostained with primary antibodies that recognize the human TIGAR protein (red signal) or high-risk subtype HPV16 E6 oncoprotein (green signal) and appropriate fluorescent secondary antibodies (Jackson ImmunoResearch Laboratories), and then analyzed by immunofluorescence-confocal microscopy at 200x magnification. Scale bar, 20 mm. DIC phase-contrast images and DAPI nuclear-staining (blue signal) are provided for reference. The lower right panel shows the 2.5 D graphical quantitation of the TIGAR-specific and HPV16 E6-specific fluorescent signals from the merged image using Carl Zeiss Microscopy Zen OS software. The data shown are representative of at least three independent experiments.

The TIGAR protein has been shown to be overexpressed in many types of cancers, including pancreatic cancer, gastric cancer, esophageal squamous cell carcinomas, nasopharyngeal carcinomas, prostate cancer, breast cancer, colorectal cancer, non-small-cell lung cancer, adult T-cell leukemia/lymphoma, acute lymphoblastic leukemia, acute myeloid leukemia, chronic lymphocytic leukemia, multiple myeloma, and malignant glioma, associated with aggressive tumor cell proliferation and poor clinical outcomes and often serves as a determinant of therapy-responsiveness [47–61]. Indeed, Zhang et al. have reported that TIGAR upregulation correlates with an aggressive disease phenotype in cervical cancer patients as determined by fluorine-18-fluorodeoxyglucose PET/computed tomography scans [62]. We therefore investigated whether the TIGAR protein is overexpressed in HPV16+ cervical cancer clinical isolates by immunofluorescence-confocal microscopy. Primary formalin-fixed and paraffin-embedded (FFPE), HPV16+ cervical carcinoma tumor sections were kindly provided by the University of Hawaii Cancer Center under a Human Subjects IRB-approved protocol. The samples were deparaffinized, immunostained using primary antibodies that recognize the human TIGAR protein and HPV16 E6 oncoprotein, and then analyzed by confocal microscopy. DAPI nuclear-staining was included to visualize all cells within the tumor microenvironment and surrounding stroma. The results from these studies revealed that the antioxidant effector TIGAR is highly expressed in HPV16+ cervical cancer clinical isolates and could prominently contribute to viral carcinogenesis by protecting rapidly proliferating tumor cells from oxidative stress and ROS-induced apoptosis (Figure 1).

### siRNA-inhibition of TIGAR protein expression sensitizes HPV18-transformed HeLa cells to low concentrations of doxorubicin

Doxorubicin (aka, adriamycin) is an anthracycline and DNA-intercollating agent that causes DNA-damage and induces ROS and is frequently used as an anticancer chemotherapeutic drug [63,64]. Cervical cancer cell-lines transformed by hrHPVs, such as HeLa and CaSki, however, exhibit significant resistance to doxorubicin-induced apoptosis [65]. Also, doxorubicin can cause clinical side-effects associated with oxidative stress, inflammation and cardiotoxicity which often limit its use as an effective anticancer agent [66,67].

**Figure 2:**
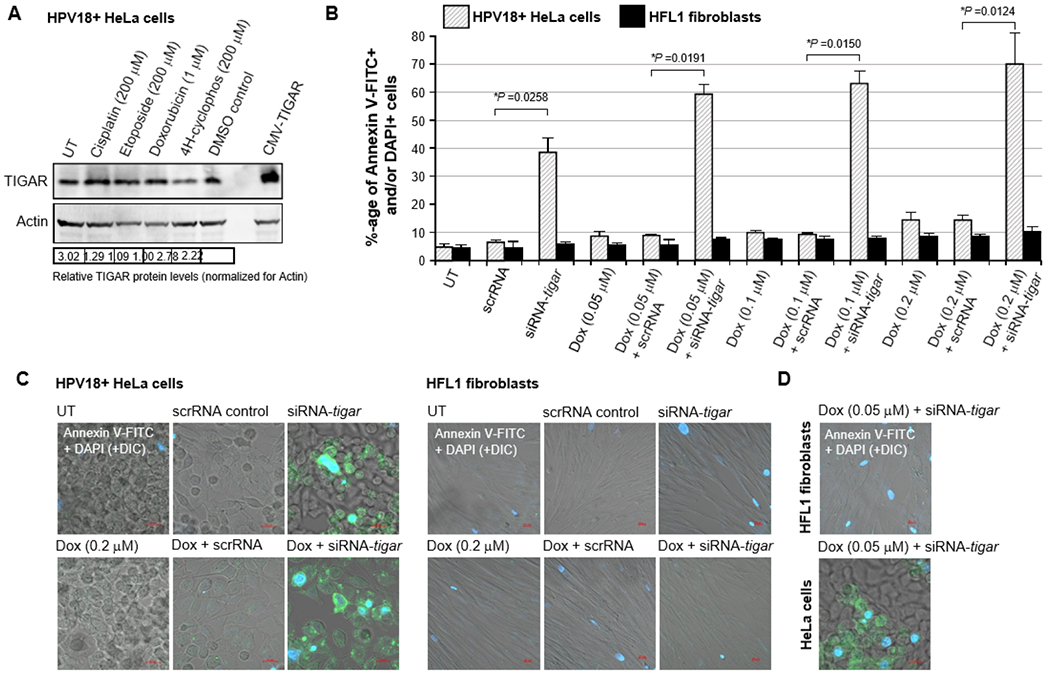
The siRNA-knockdown of TIGAR expression sensitizes HPV18-transformed HeLa cells to apoptosis induced by the chemotherapy drug, doxorubicin. A) The treatment of HPV18+ HeLa cervical adenocarcinoma cells with the chemotherapy agents, cisplatin, etoposide, doxorubicin, or 4-hydroxycyclophosphamide did not significantly alter the expression of the TIGAR protein as compared to a 1% dimethyl sulfoxide (DMSO) solvent control. HeLa cells transfected with a CMV-TIGAR expression construct are shown in the last lane for comparison [19]. The relative expression of TIGAR was assessed by SDS-PAGE and immunoblotting and quantified by densitometry with normalization to Actin protein levels. B) HPV18+ HeLa cells were grown on glass coverslips in 35 mm^2^ tissue-culture dishes and then transfected with either 25 ng of an siRNA-tigar or scrRNA oligonucleotide as a negative control. The cultures were treated with increasing concentrations of solubilized doxorubicin (Sigma-Aldrich): 0.05, 0.1, or 0.2 μM, incubated for another 24 hours, and then stained with Annexin V-FITC and DAPI and analyzed by confocal microscopy. The relative levels of cellular apoptosis were determined by quantifying the percentages of Annexin V- FITC-positive and/or DAPI-positive cells in each visual field at 200x magnification. Triplicate fields were counted for each sample. C) Representative micrographs are shown which depict the induction of apoptosis (i.e., Annexin V-FITC and/or DAPI-positive cells) in HPV18-transformed HeLa cells transfected with siRNA-tigar or siRNA-tigar+doxorubicin (0.2 μM), as compared to a scrRNA negative control or untreated (UT) cells (left panels). The effects of siRNA-tigar or the scrRNA control, either alone or in the presence of doxorubicin, in transfected human HFL1 fibroblasts are shown in the right panels. D) Representative micrographs are shown that depict the induction of apoptosis (i.e., Annexin V-FITC and/or DAPI-positive cells) in transfected HeLa cells that contain siRNA-tigar+doxorubicin (0.05 μM), as compared to HFL1 fibroblasts with siRNA-tigar+doxorubicin. DIC phase contrast is provided for the visualization of all cells within the field and for comparison in the merged images. Scale bar, 20 μm. All the data in A-D is representative of at least three independent experiments, and the data in B represent the mean of the experiments ± standard deviation (error bars).

To determine if the siRNA-knockdown of TIGAR expression could sensitize hrHPV-infected cervical cancer cells to otherwise sub-inhibitory concentrations of doxorubicin, HPV18-transformed HeLa cells or immortalized human HFL1 fibroblasts were transfected with 25 ng of either siRNA-TIGAR or a scrRNA oligonucleotide as a negative control and then, after 24 hrs, treated with various concentrations (0.05, 0.1, or 0.2 μM) of doxorubicin. The immunoblotting data in Figure 2A show that none of the chemotherapy agents used in these studies significantly altered the expression of endogenous TIGAR at the concentrations indicated, as compared to a DMSO solvent control in treated HeLa cells. The TIGAR protein expression levels were quantified by densitometry relative to Actin; and the last lane depicts cells that were transfected with a CMV-TIGAR expression construct as an antibody-staining control (Figure 2A) [19]. As shown in Figures 2B and 2C, HeLa cells were highly resistant to doxorubicin-treatment. However, the cells that were transfected with siRNA-TIGAR in combination with doxorubicin exhibited significant cytotoxicity and apoptosis (i.e., relative percentages of Annexin V-FITC-positive and/or DAPI-positive cells per field), as compared to cells transfected with the scrRNA control+doxorubicin-treatment (Figures 2B and 2C). The representative micrographs in Figure 2C depict the relative levels of apoptosis observed in HeLa cells (left panels) and HFL1 fibroblasts (right panels) transfected with either siRNA-TIGAR or a scrRNA negative control and treated with the highest concentration (0.2 μM) of doxorubicin. The images in Figure 2D similarly demonstrate that the siRNA-knockdown of TIGAR expression induced significant cytotoxicity with the lowest concentration of doxorubicin (0.05 μM) in the HPV18-transfomed HeLa cells, whereas no significant apoptosis was observed in the non-transformed HFL1 fibroblasts (Figures 2B–2D). These findings suggest that therapeutically inhibiting TIGAR functions could sensitize hrHPV-induced cancers to low doses of doxorubicin and may reduce the potential risks for chemotherapy-induced adverse side-effects, such as cardiotoxicity [66,67].

### siRNA-knockdown of TIGAR expression sensitizes HPV18-transformed HeLa cells to sub-inhibitory concentrations of the chemotherapeutic cisplatin

Platinum-based chemotherapy drugs, such as cisplatin, are indicated for the treatment of advanced-stage cervical cancers, including hrHPV-associated tumors although, by contrast, Padilla et al. have shown that hrHPV-infected cervical cancer cells are highly resistant to chemotherapeutic agents, including cisplatin [68,69]. Others have combined cisplatin treatment with photodynamic therapy and demonstrated this approach can effectively reduce the viability of hrHPV-transformed cervical cancer cell-lines *in vitro* [70]. Also, chemotherapy with cisplatin in combination with radiotherapy is routinely used for the treatment of hrHPV+ head-and-neck squamous cell carcinomas, though with mixed clinical outcomes [71]. The metallotherapeutic cisplatin induces oxidative stress and DNA-damage, ROS production, and mitochondrial membrane depolarization associated with its anti-tumorigenic cytotoxic activity [72,73]. However, platinum-based medications are often also associated with adverse clinical side-effects, including nephrotoxicity and acute kidney damage, intestinal injury, hearing loss, as well as chemotherapy-induced peripheral neuropathies [74–78]. Thus, as a long-term goal, it would be beneficial to therapeutically hypersensitize cancer cells to reduced concentrations of cisplatin to lower its effective dosage and minimize the potential risks of secondary tissue damage by this metallo-chemotheraputic agent.

**Figure 3:**
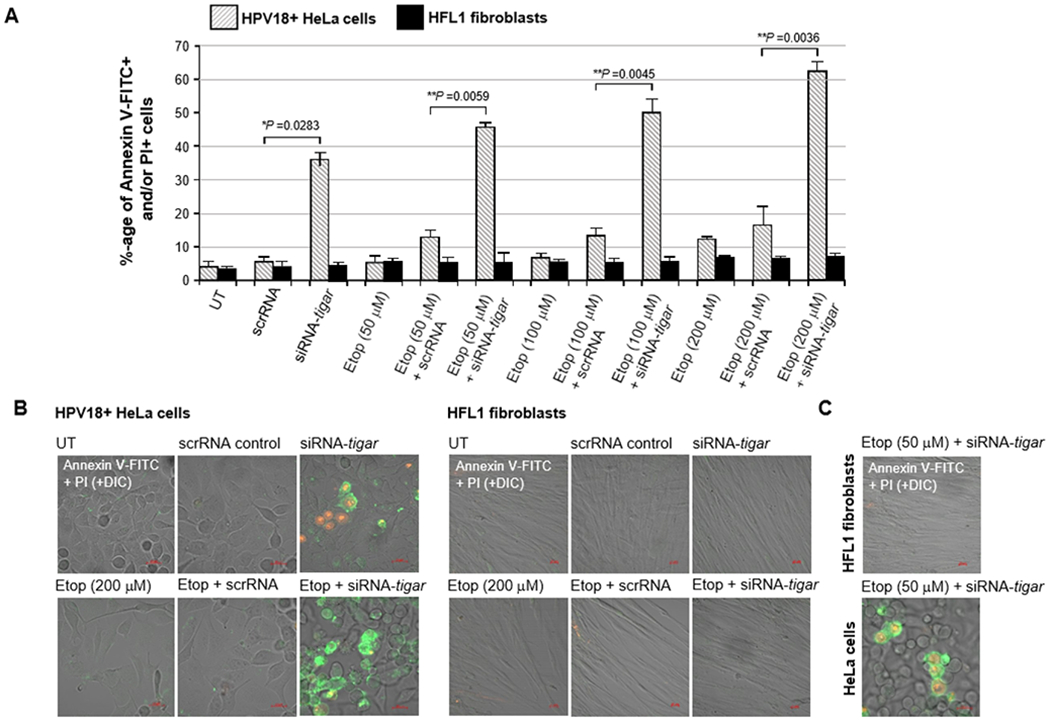
siRNA-inhibition of TIGAR expression sensitizes HPV18-transformed HeLa cells to apoptosis induced by low concentrations of cisplatin. A) HPV18+ HeLa cells were transfected with 25 ng of siRNA-tigar or a scrRNA oligonucleotide as a negative control. The cells were then treated with increasing concentrations of cisplatin (Sigma-Aldrich): 0.01, 0.05, or 0.1 μM, incubated for another 24 hours, and stained with Annexin-V-FITC and PI and the relative percentages of apoptotic (i.e., Annexin V-FITC-positive and/or PI-positive) cells in each visual field were quantified at 200x magnification by confocal microscopy. Triplicate fields were counted for each sample. B) Representative micrographs are shown which depict the induction of apoptosis (i.e., Annexin V-FITC and/or PI-positive cells) in HPV18+ HeLa cells transfected with siRNA-tigar or siRNA-tigar+cisplatin (0.1 μM), as compared to a scrRNA negative control or untreated (UT) cells (left panels). The effects of siRNA-tigar or the scrRNA control, either alone or in the presence of cisplatin, in transfected human HFL1 fibroblasts are shown at right. C) The representative micrographs shown depict the induction of apoptosis in transfected HeLa cells that contain siRNA-tigar+cisplatin (0.01 μM), as compared to HFL1 fibroblasts with siRNA-tigar+cisplatin. DIC phase contrast is provided for the visualization of all cells within the field and for comparison in the merged images. Scale bar, 20 μm. All the data is representative of at least three independent experiments, and the data in A represent the mean of the experiments ± standard deviation (error bars).

To determine whether the inhibition of TIGAR expression by siRNA-TIGAR could sensitize HPV18+ HeLa cells to cisplatin-induced cytotoxicity, the cultures were grown on glass coverslips and transfected with 25 ng of an siRNA-TIGAR oligonucleotide or scrRNA as a negative control. Then, after 24 hours, the cells were treated with various concentrations of solubilized cisplatin: 0.01, 0.05, or 0.1 μM. Following another 24 h, the samples were stained with Annexin V-FITC and PI and subsequently analyzed by fluorescence-confocal microscopy to quantify the relative levels of cellular apoptosis by measuring the percentages of Annexin V-FITC-positive and/or PI-positive cells per field at 200x magnification. As shown in Figure 3A, the HeLa cultures were largely insensitive to cisplatin-induced apoptosis, compared to untreated cells or the scrRNA control. The siRNA-inhibition of TIGAR expression was observed to induce significant programmed cell-death (approximately 42%) in transfected HeLa cells. Moreover, the combination of siRNA-TIGAR with increasing amounts of the drug cisplatin resulted in higher levels of apoptosis, as compared to cisplatin treatment with the scrRNA negative control (Figure 3A). The representative micrographs in the left panels of Figure 3B show markedly higher numbers of Annexin V-FITC (green) and PI (red)-positive cells in the samples treated with either siRNA-TIGAR alone or siRNA-TIGAR+cisplatin (0.1 μM), as compared to the scrRNA control+cisplatin (0.1 μM). Neither siRNA-TIGAR alone nor siRNA-TIGAR+cisplatin (0.1 μM) caused detectable levels of apoptosis in transfected HFL1 fibroblasts (Figures 3A and 3B, right panels). The micrographs in Figure 3C demonstrate that the combination of siRNA-TIGAR with the lowest concentration of cisplatin (0.01 μM) resulted in significant cytotoxicity and apoptosis in transfected HeLa cells, as compared to HFL1 fibroblasts. These findings suggest that the siRNA-knockdown of TIGAR expression sensitizes HPV18-transformed HeLa cervical carcinoma cells to oxidative DNA-damage and cytotoxicity induced by the metallo-chemotherapeutic cisplatin.

siRNA-knockdown of TIGAR expression sensitizes HPV18+ HeLa cells to reduced concentrations of the topoisomerase II-inhibitor, etoposide

The chemotherapeutic agent, etoposide, inhibits topoisomerase II complexes and is frequently used in anticancer treatment regimens against recurrent or primary advanced cervical carcinomas, often in combination with radiotherapy [79,80]. Reddy et al. have also reported that stabilization of the p53 tumor suppressor protein in HeLa cells sensitizes them to etoposide-induced cellular arrest and apoptosis [81]. However, high doses of etoposide have been associated with liver toxicity and nephrotoxicity as complicating clinical side-effects [82,83].

**Figure 4:**
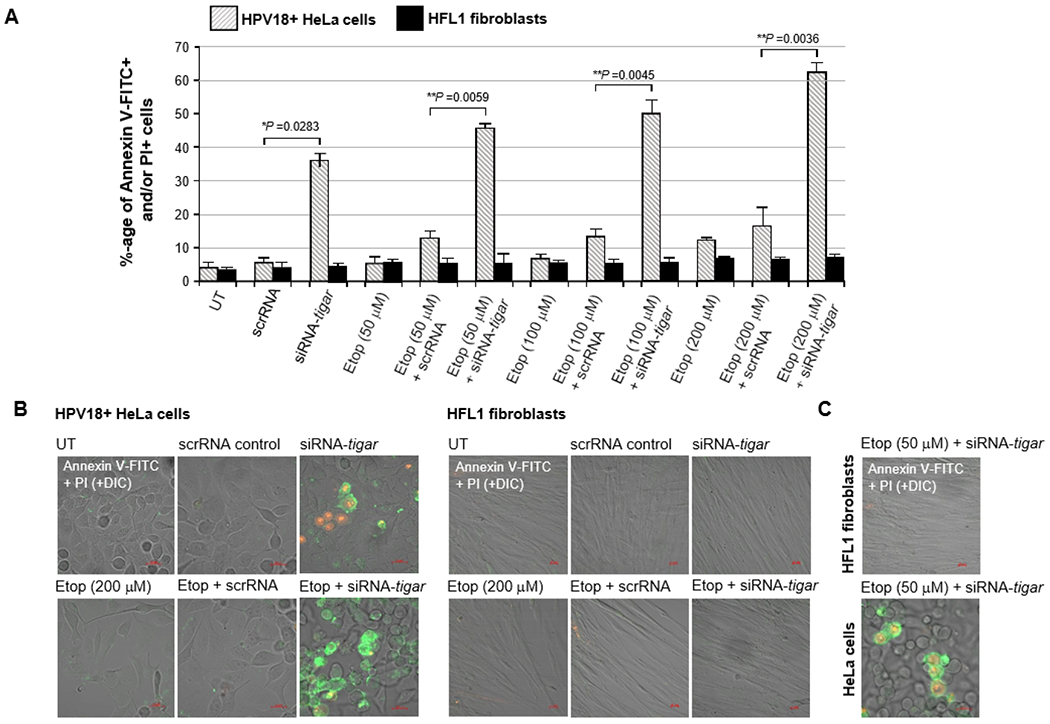
The siRNA-knockdown of TIGAR expression sensitizes HPV18-transformed HeLa cells to apoptosis induced by the genotoxic chemotherapy agent, etoposide. A) HPV18+ HeLa cells and human HFL1 fibroblasts were grown on glass coverslips in 35 mm^2^ tissue cultures dishes and then transfected with 25 ng of either siRNA-tigar or a scrRNA oligonucleotide as negative control. The transfected cells were treated with an increasing concentration of etoposide (Sigma-Aldrich): 50, 100, or 200 μM, and incubated for another 24 hours. The cells were then stained with Annexin V-FITC and PI and the relative percentages of apoptotic (i.e., Annexin V-FITC-positive and/or Pi-positive) cells in each visual field were quantified at 200x magnification using confocal microscopy. Triplicate fields were counted for each sample. B) Representative micrographs that depict the induction of apoptosis in HPV18+ HeLa cells transfected with siRNA-tigar or a scrRNA control and treated with etoposide (200 μM), as compared to untreated (UT) cells, are shown in the left panels. Human HFL1 fibroblasts transfected with siRNA tigar or a scrRNA control, either alone or in combination with etoposide-treatment, are shown at right. C) Representative micrographs depict the induction of apoptosis in HeLa cells transfected with siRNA-tigar and then treated with the lowest concentration of etoposide (50 μM), as compared to human HFL1 fibroblasts. DIC phase contrast is provided for the visualization of all cells within the field and for comparison in the merged images. Scale bar, 20 μm. All the data is representative of at least three independent experiments, and the data in A represent the mean of the experiments ± standard deviation (error bars).

Thus, to determine if inhibiting TIGAR could sensitize hrHPV+ cervical carcinoma cells to reduced concentrations of etoposide, HeLa cells were cultured on glass coverslips and then transfected with 25 ng of either siRNA-TIGAR or a scrRNA oligonucleotide as a negative control. The cultures were subsequently treated with increasing concentrations of an etoposide solution: 50, 100, or 200 μM. In parallel, immortalized (non-transformed) HFL1 fibroblasts ere transfected and treated with various concentrations of the drug for comparison. The samples were then stained with Annexin V-FITC and PI and the relative percentages of apoptotic cells per field were quantified using confocal fluorescence-microscopy. As shown in Figure 4A, although the siRNA-inhibition of TIGAR expression induced moderate cytotoxicity in the HPV18+ HeLa cells, no significant apoptosis was observed in HFL1 fibroblasts transfected with the siRNA-TIGAR oligonucleotide. The scrRNA control did not cause cytotoxicity in either the transfected HeLa or HFL1 cells (Figure 4A). The representative micrographs in Figure 4B demonstrate that siRNA-TIGAR alone, as well as siRNA-TIGAR+etoposide (200 μM), resulted in significant cytotoxicity (i.e., Annexin V-FITC-positive and/or PI-positive cells/field) as compared to the scrRNA control+etoposide-treatment. The right panels in Figure 4B demonstrate that neither siRNA-TIGAR nor siRNA-TIGAR+etoposide (200 μM) caused any detectable cellular apoptosis in the non-transformed HFL1 fibroblast cell-line. The data in Figure 4C further show that siRNA-TIGAR-transfected HPV18+ HeLa cells exhibited significant cytotoxicity and apoptosis even with the lowest (otherwise sub-inhibitory) concentration (50 μM) of etoposide. These results suggest that therapeutically inhibiting TIGAR could make hrHPV-induced cervical cancers hypersensitive to low concentrations of etoposide and thereby reduce the potential for adverse side-effects associated with this chemotherapeutic agent.

### siRNA-knockdown of TIGAR expression counters the resistance of HPV18+ HeLa cells to the DNA-alkylating drug, cyclophosphamide

Others have previously reported that the HPV18+ HeLa cervical carcinoma cell-line is highly resistant to cytotoxicity and apoptosis induced by the 7-guanine-alkylating (i.e., nitrogen mustard family) chemotherapeutic agent, cyclophosphamide [84,85]. This compound functions as a prodrug which is metabolized by hepatic enzymes (e.g., cytochrome P450s) into its active form, 4-hydroxycyclophosphamide, together with the tautomer aldophosphamide, which are spontaneously converted into a DNA-alkylating phosphoramide mustard that modifies the 7-N position of guanine [86]. Cyclophosphamide and its metabolites have also been shown to induce oxidative stress and DNA-damage through the production of intracellular ROS and nitric oxide resulting in the generation of toxic peroxynitrite in rapidly proliferating cells [87]. While cyclophosphamide is often used in combination chemotherapy regimens to treat hematological malignancies, including leukemias, Hodgkin’s and non-Hodgkin’s lymphomas, and multiple myeloma, as well as many carcinoid tumors, such as small-cell lung cancers and ovarian cancer, it is not indicated for use against hrHPV+cervical cancers [88,89]. This alkylating agent has also been linked with severe clinical toxicity affecting the bone marrow, reproductive organs, heart, kidneys and liver which limits its anticancer therapeutic applications [84,90–93].

**Figure 5:**
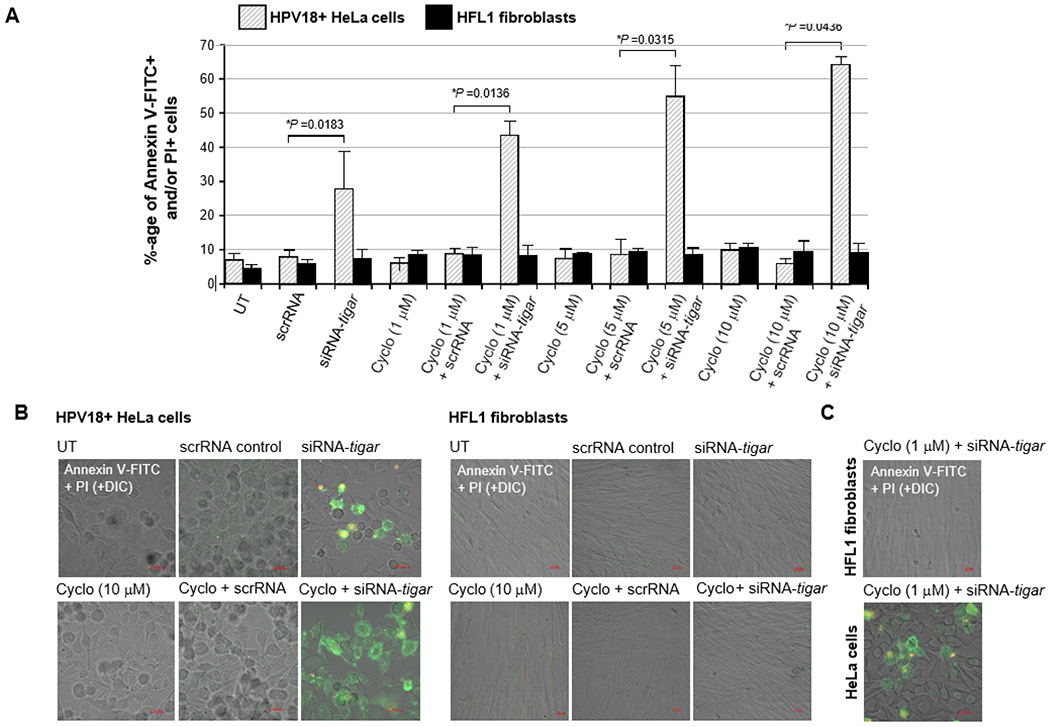
siRNA-inhibition of TIGAR expression sensitizes HPV18-transformed HeLa cells to the active metabolite of the alkylating chemotherapy drug, 4-hydroxycyclophosphamide. A) HPV18+ HeLa cervical adenocarcinoma cells and human HFL1 fibroblasts were grown on glass coverslips and then transfected with 25 ng of either siRNA-tigar or a scrRNA oligonucleotide as negative control. The cultures were treated with increasing concentrations of 4-hydroxycyclophosphamide (Sigma-Aldrich): 1, 5, or 10 μM, and then incubated for another 24 hours. The samples were then stained with Annexin V-FITC and PI and analyzed by confocal microscopy. The relative percentages of apoptotic (i.e., Annexin V-FITC- positive and/or PI-positive) cells in each visual field were quantified by counting at 200x magnification. Triplicate fields were counted for each sample. B) The representative micrographs at left depict the induction of apoptosis in HPV18+ HeLa cells transfected with siRNA-tigar or a scrRNA control and treated with the highest concentration (10 μM) of 4- hydroxycyclophosphamide. Untreated (UT) cells are shown for comparison. HFL1 fibroblasts transfected with siRNA-tigar or the scrRNA negative control and treated with 4- hydroxycyclophosphamide are shown at right. C) Representative micrographs of HeLa cells and human HFL1 fibroblasts that were transfected with siRNA-tigar or a scrRNA control and treated with the lowest concentration (1 μM) of 4-hydroxycyclophosphamide are shown. DIC phase contrast is provided for the visualization of all cells within the field and for comparison in the merged images. Scale bar, 20 μm. All the data is representative of at least three independent experiments, and the data in A represent the mean of the experiments ± standard deviation (error bars).

To determine whether the siRNA-inhibition of TIGAR expression could sensitize HPV18+ HeLa cells to oxidative DNA-damage induced by cyclophosphamide, the cells were cultured on glass coverslips and then transfected with siRNA-TIGAR or a scrRNA oligonucleotide as a negative control. After 24 hours, the cells were treated with increasing concentrations of the bioactive metabolite 4-hydroxycyclophosphamide: 1, 5, or 10 μM, and subsequently analyzed for the induction of apoptosis by staining them with Annexin V-FITC/PI, followed by confocal microscopy imaging. The results in Figure 5A demonstrate that siRNA-TIGAR+4-hydroxycyclophosphamide-treatment resulted in significant cytotoxicity and cellular apoptosis which increased in a dose-dependent manner. This was not observed with the combination of scrRNA+4-hydroxycyclophosphamide. Also, we did not observe any apoptosis in the non-transformed HFL1 fibroblasts that were transfected with siRNA-TIGAR and/or treated with 4-hydroxycyclophosphamide (Figures 5A and 5B). The representative micrographs in Figure 5B demonstrate that a significantly higher number of Annexin V-FITC and/or PI-positive HeLa cells were detected in the siRNA-TIGAR+4-hydroxycyclo-phosphamide (10 μM)-treated samples, as compared to siRNA-TIGAR alone (left panels). Further, the micrographs in Figure 5C demonstrate that siRNA-inhibition of TIGAR expression sensitized the HPV18+ HeLa cell-line to the lowest concentration of 4-hydroxycyclophosphamide. These aggregate findings suggest that inhibiting TIGAR functions could potentially hypersensitize hrHPV-associated cervical cancer cells to DNA-damage and cytotoxicity induced by chemotherapeutic agents that cause oxidative stress, and may sensitize these tumor cells to cyclophosphamide-a phosphoramide DNA-alkylating drug that is not presently indicated for use against hrHPV+ cervical cancers and which Hela cells are known to have resistance to.

## DISCUSSION

The myriad and complex molecular mechanisms by which oncogenic viruses deregulate cellular growth and proliferative pathways to cause human cancers remain be completely defined. The scope of the present study has been to determine whether inhibiting expression of the TIGAR protein-a p53-regulated glycolytic enzyme and antioxidant effector [19,20,46], could hypersensitize hrHPV-transformed cervical carcinoma cells to cytotoxicity induced by chemotherapy drugs (i.e., cisplatin, etoposide, doxorubicin, and 4-hydroxycyclophosphamide) that cause oxidative stress and DNA-damage [63,64,72,73,86,87]. The hrHPVs infect basal stem cells of the epidermis and establish latent infections-with viral replication and the expression of the regulatory proteins E1, E2, E4, E5, E6 and E7 linked to differentiation programming in keratinized epithelial cells [25]. Mature virus particles are assembled within the stratified spinous and granular layers and then shed from the outermost corneum of the skin [25]. The hrHPVs cause neoplastic diseases in 3%-5% of infected individuals and have been etiologically linked with cervical dysplasia and carcinomas, vulvar, vaginal, penile and anogenital cancers, as well as head-and-neck cancers [10–17]. While there are two multivalent preventative vaccines available which target many of the major hrHPVs, including HPV16, HPV18, HPV31, HPV33, HPV45, HPV52, and HPV58, there nevertheless persists a significant incidence of hrHPV-induced cancers-with anogenital squamous cell carcinomas, in particular, on the rise in recent years in HIV-positive and-negative populations [18,94,95]. There have also been increasing incidences of oropharyngeal cancers associated with HPV infections [96].

The viral E6 oncoprotein cooperates with cellular oncogenes (e.g., c-Myc and Ha-Ras) and transcriptionally activates the htert promoter associated with cellular immortalization [36,37]. Further, E6 interacts with the E3 ubiquitin ligase, E6AP, and destabilizes the p53 tumor suppressor by promoting the continuous turnover of this gatekeeper protein [38–40]. Surprisingly, however, although p53 is mutated or functionally inactivated in nearly half of all cancers, p53 genetic mutations rarely occur in hrHPV+ clinical isolates, suggesting that p53-regulated target gene products could contribute to viral carcinogenesis [45]. The E6 oncoprotein has also been shown to reduce intracellular levels of the TIP60 acetyltransferase through modulation of the E3 ligase, EDD1, and inhibit K120-acetylation of the p53 protein and prevent the induction of p53-dependent proapoptotic genes [41,97]. Most hrHPV+ cervical cancer cell-lines, including HeLa and CaSki, contain detectable levels of p53; and mounting evidence has further demonstrated that the remaining p53 protein present within HPV-infected cells is transcriptionally active [42–44]. Also, the p53-regulated antioxidant effector TIGAR is highly expressed in the E6-positive tumor cells of hrHPV+ cervical cancer clinical isolates (Figure 1). We therefore tested whether TIGAR contributes to the resistance of HPV18+ HeLa adenocarcinoma cells to chemotherapy agents that induce oxidative stress. None of the drugs used for these experiments altered the expression of the endogenous TIGAR protein, compared to a DMSO solvent control, as determined by immunoblotting (Figure 2A). Although the siRNA-tigar oligonucleotide induced moderate levels of cytotoxicity in transfected HeLa cells, we did not observe any significant cellular apoptosis in the immortalized HFL1 fibroblasts either by siRNA-tigar or the scrRNA negative control (Figures 2A–2C, 3A–3B, 4A–4B, and 5A–5B). The scrRNA was not cytotoxic in transfected HeLa cells.

We next tested whether siRNA-knockdown of TIGAR expression could hypersensitize HPV18+ HeLa cervical carcinoma cells to otherwise sub-inhibitory concentrations of the chemotherapeutic agents: doxorubicin, cisplatin, etoposide, or 4-hydroxycyclophosphamide. Of these drugs, none caused significant cellular toxicity over the ranges of concentrations tested in the treated HeLa or HFL1 cultures; and only etoposide exhibited a slight increase in apoptosis in HeLa cells transfected with the scrRNA control oligonucleotide (Figures 2A, 3A, 4A, and 5A). This effect was not observed in the etoposide-treated HFL1 fibroblasts transfected with scrRNA (Figures 4A and 4B). By contrast, the siRNA-TIGAR-transfected HeLa cells exhibited markedly enhanced cytotoxicity and dosage-dependent apoptosis with increasing concentrations of each of the chemotherapy agents tested in these studies (Figures 2B–2D, 3A–3C, 4A–4C, and 5A–5C). This cooperativity was not observed, however, in the transfected HFL1 cells. These results demonstrate that inhibiting TIGAR expression enhances the oxidative stress and damaging effects of ROS produced by anticancer chemotherapy drugs in virus-transformed cervical tumor cells. Many of these compounds are known to cause severe toxicity and damage to other tissues, including the liver, kidneys, heart, bone marrow, reproductive organs, and nervous system, when used at higher doses which can limit their therapeutic applications [66,67,74–78,82–84,90–93].

## CONCLUSION

In summary, our findings suggest that therapeutically inhibiting TIGAR could represent a plausible strategy to hypersensitize hrHPV+ tumor cells to otherwise sub-inhibitory concentrations of chemotherapy agents that cause oxidative stress and DNA-damage and, consequently, reduce the risks of adverse clinical side-effects associated with these anticancer medications. These data further imply that inhibiting TIGAR sensitizes HPV18+ cervical cancer cells to the DNA-alkylating drug, cyclophosphamide and thus could add yet another therapeutic to the arsenal against virus-induced cancers.
